# Metabolic enzyme clustering by coiled coils improves the biosynthesis of resveratrol and mevalonate

**DOI:** 10.1186/s13568-020-01031-5

**Published:** 2020-05-24

**Authors:** Tina Fink, Bojana Stevović, René Verwaal, Johannes A. Roubos, Rok Gaber, Mojca Benčina, Roman Jerala, Helena Gradišar

**Affiliations:** 1 Department of Synthetic Biology and Immunology, National Institute of Chemistry, Hajdrihova 19, 1000 Ljubljana, Slovenia; 2 DSM Biotechnology Center, DSM, Delft, The Netherlands; 3 EN-FIST Centre of Excellence, Ljubljana, Slovenia

**Keywords:** Designed coiled-coil dimers, Enzyme clustering, Biosynthesis, Resveratrol, Mevalonate

## Abstract

The clustering of biosynthetic enzymes is used in nature to channel reaction products and increase the yield of compounds produced by multiple reaction steps. The coupling of multiple enzymes has been shown to increase the biosynthetic product yield. Different clustering strategies have particular advantages as the spatial organization of multiple enzymes creates biocatalytic cascades with a higher efficiency of biochemical reaction. However, there are also some drawbacks, such as misfolding and the variable stability of interaction domains, which may differ between particular biosynthetic reactions and the host organism. Here, we compared different protein-based clustering strategies, including direct fusion, fusion mediated by intein, and noncovalent interactions mediated through small coiled-coil dimer-forming domains. The clustering of enzymes through orthogonally designed coiled-coil interaction domains increased the production of resveratrol in *Escherichia coli* more than the intein-mediated fusion of biosynthetic enzymes. The improvement of resveratrol production correlated with the stability of the coiled-coil dimers. The coiled-coil fusion-based approach also increased mevalonate production in *Saccharomyces cerevisiae*, thus demonstrating the wider applicability of this strategy.

## Introduction

The biosynthesis of complex compounds involves several enzyme catalysts, which need to act consecutively on the reaction substrates and intermediates to produce the desired end product. Natural enzymes are frequently organized into clusters, such as in large enzyme complexes (Conrado et al. 2008) or in microcompartments (Colin et al. 2015; Polka et al. 2016). This offers several benefits, including increased production yield, decreased toxicity by intermediates, and decreased side-reaction metabolic flux. Typically, biosynthetic reactions performed in the host chassis by multiple ectopically expressed enzymes from different organisms have not been optimized; in such cases, however, enzyme clustering may increase the production yield (Jandt et al. 2013; Castellana et al. 2014; Ji et al. 2016). Several strategies have been developed and have been reported to achieve enzyme clustering, including covalent enzyme fusions (Zhang et al. 2006;), noncovalent assembly through the fusion of enzyme to protein–protein interaction domains (Dueber et al. 2009; Diener et al. 2016), the fusion of enzymes to DNA- or RNA-binding domains (in combination with DNA/RNA scaffolds) (Delebecque et al. 2011; Conrado et al. 2012; Sachdeva et al. 2014), and the coupling of enzymes to oligonucleotides that direct them into the designed DNA nano-scaffolds (Ke et al. 2016). However, for the spatial organization of a broad range of enzymes in desirable and optimal microenvironments for reaction sequences, a versatile protein scaffolds from natural and unnatural protein, and peptide domains have been extensively studied in last decade (Lee et al. 2012; Wang and Yu 2012; Yang et al. 2017; Li et al. 2018; Schmidt-Dannert et al. 2018; Zhang et al. 2018, 2019; Lim et al. 2019).

Each clustering strategy has certain advantages and drawbacks. These can depend on the specific reactions and enzymes in question. Additionally, the efficiency of each strategy may depend on the host chassis since different cellular environments (such as the reducing potential and the presence of folding chaperones or other cellular components) may affect the efficiency of each strategy. To minimize the metabolic burden of synthesizing new proteins in the production organism, the clustering domains should be small in size, and, above all, they should not interfere with the other host cellular components. Here, we have compared different enzyme-clustering strategies for the biosynthesis of resveratrol and mevalonate, both commercially interesting products. The use of clustering to increase the production of resveratrol and mevalonate was investigated prior to the use of several different approaches (Halls and Yu 2008; Dueber et al. 2009; Conrado et al. 2012) and can be said, therefore, to represent an appropriate benchmarking model. We anticipated that utilizing a strategy of enzyme clustering via designed coiled-coil interaction domains would avoid problems connected to the folding of multi-enzyme complexes. Our previous studies revealed the orthogonality of de novo designed coiled coils (Gradišar and Jerala 2011; Drobnak et al. 2017), which are suitable for the fusion of small coiled-coil-forming domains to different enzymes on selected biosynthetic pathways. We demonstrate the efficiency of this method in increasing production yields for resveratrol in bacteria. Finally, the study also finds that coiled-coil interaction-based clustering improves the biosynthesis of mevalonate in yeast, thus demonstrating the wider applicability of this strategy.

## Materials and methods

### Construction of plasmids

The enzymes with N-terminal His-tag from a resveratrol biosynthetic pathway, 4-coumaroyl-CoA ligase (4CL) from *Arabidopsis thaliana,* and stilbene synthase (STS) from *Vitis vinifera* (Wang et al. 2011) were codon optimized for expression in *E. coli* and cloned into a pET19b vector. The N- and C-terminal intein fragments (IntN and IntC) of the NpuDnaE intein for covalent intein fusion were obtained from the pSKDuet01 (Addgene plasmid # 12172). The direct fusion of 4CL and STS with a 10-amino acid residue linker and N-terminal His-tag was cloned into pET19b. The fusions of 4CL and STS with different coiled-coil-forming domains (CC), C-terminally fused to 4CL and STS with N-terminal His-tag, were cloned into pET19b. The co-expression of plasmids pET19b-(4CL-STS), pET19b-(4CL:intN-intC:STS), and pET19b-(4CL:CC-STS:CC) contained a tandem of ^*His*^*4CL* and ^*His*^*STS*, ^*His*^*4CL:intN* and ^*His*^*intC:STS,* or ^*His*^*4CL:CC* and ^*His*^*STS:CC* genes (each under the control of a separate T7 promoter, RBS site, and T7 terminator), respectively.

### Bacterial strains, media, and growth conditions for resveratrol production

*Escherichia coli* strain BL21(DE3)pLysS (Invitrogen) was transformed with respective constructs and grown at 37 °C overnight (160 rpm) in LB medium for inoculum preparation. Bacterial cultures were transferred to 100 mL of fresh 2YT medium with antibiotics (ampicillin and chloramphenicol at concentrations of 50 µg/mL and 34 µg/mL, respectively) and grown until OD_600_ reached 0.6. After induction with 0.7 mM IPTG, cultures were grown for an additional 3 h at 37 °C. The harvested cells were resuspended in minimal M9 medium with antibiotics, 0.1 mM IPTG, and 1 mM p-coumaric acid as a substrate for resveratrol production. Fermentation continued at 25 °C for 20 h (160 rpm), and resveratrol production was analyzed by liquid chromatography-tandem mass spectrometry (LC–MS/MS) after 1.5 h, 4 h, 6 h, 8 h, and 20 h.

### Resveratrol LC–MS/MS analysis

Resveratrol quantification was performed by an LC–MS/MS analysis of the supernatant. The supernatant from 1 mL of fermentation broth was acidified with 6.25 µL of 1 M HCl and heated to 95 °C for 5 min. The sample was centrifuged at 17,000 rpm (10 min), and 600 µL of the sample was analyzed using a Shimadzu Prominence Liquid Chromatographic system with the Accucore aQ 100 mm × 2.1 mm, 2.6 µm column (Thermo Scientific) at 30 °C. The sample (2 µL) was separated at a flow rate of 400 µL/min using a linear acetonitrile (MeCN)/water gradient from 2% B to 70% B within 4.4 min (solvent A: 1% MeCN, 99% H2O, 0.2% FA (formic acid); solvent B: 99% MeCN, 1% H2O, 0.2% FA). MS detection with an ESI source (Shimadzu LC-MS8030 triple quadruple) was performed in positive ion mode in N2 flow with a detector voltage of 4.5 kV. Quantification was performed in the multiple reaction monitoring (MRM) mode in a tandem mass spectrometer MS/MS to detect transitions *(m/z)*: for resveratrol 229 → 107, 134, 91; for p-coumaric acid 165 → 146, 91, 64.

### Western blotting analysis

Cells from 5 mL of fermentation broth were resuspended in 350 µL of lysis buffer (100 mM Tris at pH 8.0, 100 mM EDTA, 100 mM MgCl2, 1 mg/mL Lysozyme (Sigma-Aldrich), 15 U/mL Benzonase (Millipore), CPI protease inhibitors (Sigma-Aldrich)) and lysed at 25 °C for 1 h. Proteins (20 µL of clear lysate) were separated on 12% SDS PAGE (sodium dodecyl sulfate–polyacrylamide gel electrophoresis) gels, blotted onto a Hybond ECL nitrocellulose membrane (GE Healthcare), and detected with mTetra-His antibodies (Qiagen) and goat anti-mouse HRP-conjugated antibodies (Jackson ImmunoResearch). ECL Western blotting detection reagent (GE Healthcare) was used for detection.

### Protein purification and native PAGE

*Escherichia coli* BL21(DE3)pLysS strain was grown in an LB medium with antibiotics at 37 °C until OD_600_ reached 0.6 and then induced with 1 mM IPTG and grown at 25 °C for an additional 6 h. The harvested cells were resuspended in a lysis buffer on ice (100 mM Tris at pH 8.0, 50 mM NaCl, 10% (w/v) glycerol, 1 mg/mL Lysozyme, 15 U/mL Benzonase, CPI protease inhibitors). Cell lysis was completed by sonication on ice for 2 min (5 s pulses) and then centrifuged at 12,000 rpm (4 °C) for 10 min. The supernatant was filtered through a 0.22 µm filter (Sartorius) and applied to the Ni–NTA column (Agarose Bead Technologies) for His-tagged-protein affinity isolation according to the manufacturer’s protocol. Eluted fractions were collected and dialyzed against a 50 mM Tris (pH 8.0) buffer containing 50 mM NaCl and 10% (w/v) glycerol.

The isolated proteins were analyzed by native PAGE. The enzyme mixtures of 4CL and STS, with or without fused coiled-coil-forming domains (0.8 mg/mL each), were prepared in a 1:1 molar ratio and incubated overnight at 4 °C or 25 °C. After the addition of native PAGE loading dye, the samples were separated on 8% separating gel at 100 V and 4 °C for 3 h. The gel was stained using the Coomassie Brilliant Blue R-250.

### Co-isolation of proteins

To confirm the interaction between two coiled-coil-forming domains C-terminally fused to 4CL and STS (^His^4CL:P3S, ^AU^STS:P4S), the co-isolation of recombinantly co-expressed proteins was performed on an Ni–NTA affinity column. As a negative control, ^His^4CL and ^AU^STS were used. After isolation, the samples were analyzed on 10% SDS PAGE gel.

### Yeast strain construction

The plasmids used for the expression of Cas9 and the sgRNA to construct the carotenoid producing *S. cerevisiae* strain named CAR-0002 (Verwaal et al. 2018) were removed from the strain by liquid transfers on a YEP medium with 2% glucose (YEPD) and selections on YEPD agar plates with and without antibiotics. The open reading frames (ORFs) encoding the enzymes of the mevalonate biosynthetic pathway (acetyl-CoA acetyl transferase (ERG10), HMG-CoA synthase (HMGS), and truncated HMG-CoA reductase (tHMGR)) with or without an N-terminally fused P3:GCN:P4 coiled-coil-forming domain were codon optimized for *S. cerevisiae* expression and ordered as synthetic DNA (ATUM). The ORFs were assembled into full expression cassettes by Golden-Gate cloning with promoter and terminator sequences and flanked by connector sequences as described by Roubos et al. (2013). A schematic representation of the expression cassettes is shown in Additional file 1: Fig. S1.

The (P3:GCN:P4)-ERG10, (P3:GCN:P4)-HMGS, and (P3:GCN:P4)-tHMGR expression cassettes were introduced into the genomic DNA of the carotenoid-producing yeast strain, which was constructed using the CRISPR/Cas9 approach described by Verwaal et al. (2018). The donor DNA cassettes were directed to the INT59 integration site, a non-coding region between *SRP40 (YKR092C)* and *PTR2 (YKR093W)* located on chromosome XI. The genomic target sequence for the sgRNA was AGAAAACTCTTAGCTTTTCC. Transformants were checked for the correct integration of the expression cassettes by PCR. Prior to growth experiments, transformants were cured from applied CRISPR-editing plasmids by liquid transfers on YEP 2% glucose. All sequences used are available upon request.

### Yeast mevalonate and carotenoid production experiment

Yeast cells were inoculated in a shake flask containing 20 mL of Verduyn medium with 2% glucose (Verduyn et al. 1992) and cultivated 48 h at 30 °C and 250 rpm. One mL of the culture was transferred to a shake flask containing 50 mL of Verduyn medium with 2% glucose and cultivated an additional 72 h.

### Mevalonate LC–MS/MS analysis

Extracellular mevalonate quantification was performed by LC–MS/MS analysis. After 72 h of growth, 1 mL of fermentation broth was centrifuged to remove cells. The supernatant was diluted 100 times with milliQ water. Samples were analyzed by using a Waters iClass UPLC Liquid Chromatographic system with a Waters Acquity HSS T3 150 mm × 2.1 mm, 1.8 µm column at 60 °C. An autosampler was set to 10 °C. A sample (2 µL) was separated at a flow rate of 400 µL/min. An acetonitrile/ammonium acetate elution started for 2 min at 100% A (solvent A: 25 mM ammonium acetate) followed by a linear gradient to 95% B (solvent B: 100% MeCN) for 1 min and by a linear gradient to 50% B for 3 min. MS/MS analysis was carried out using the Waters Xevo TQD triple quadruple mass spectrometer. MS detection with an ESI source was performed in negative ionization mode (capillary voltage 2 kV, cone voltage 20 V, collision energy 10 V, desolvation temperature 600 °C, source temperature 150 °C). Quantification was performed in the MRM mode in a tandem mass spectrometer MS/MS to detect transitions *(m/z)* for mevalonate 147 → 86.6, 58.8.

### Carotenoid measurement

Carotenoids were extracted using a PRECELLYS^®^ 24 tissue homogenizer. An equivalent of 20 OD_600_ units (e.g., 2 mL with OD_600_ of 10) of culture was pelleted in a PRECELLYS tube, and the pellet was extracted with 1 mL of tetrahydrofuran (containing 0.01% butylhydroxytoluene) by homogenization for 3 × 15 s at 6500 rpm. Following 5 min of centrifugation at 4 °C, 800 µL was transferred to a glass vial. Extracts were dried down and resuspended in 80 µL dichloromethane followed by 720 µL of a 50%:50% (v/v) mixture of heptane and ethyl acetate (containing 0.01% butylhydroxytoluene). A high-performance liquid choromatography (HPLC) analysis of carotenoids was performed, as described previously (Bailey et al. 2010).

### Nucleotide sequence accession numbers

The nucleotide sequences of the codon-optimized synthetic genes for resveratrol production in *E. coli* were deposited at the Addgene database under accession numbers 139789, 139790, 139791, 139792, 139793, 139794, 139795, 139796, 139797, 139798, and 139799. The nucleotide sequences of the codon-optimized synthetic genes for mevalonate production in *S. cerevisiae* were deposited at the NCBI GenBank database under accession numbers 2308555, 2309511, 2309514, 2309522, 2309560, 2309561, 2309563, 2309567, and 2309569. The nucleotide sequences of the Cas9 plasmid and the sgRNA were deposited at the Addgene database under accession numbers 101725 and 101750, respectively (Verwaal et al. 2018).

## Results

### Evaluating strategies of covalently fused enzymes

**Fig. 1:**
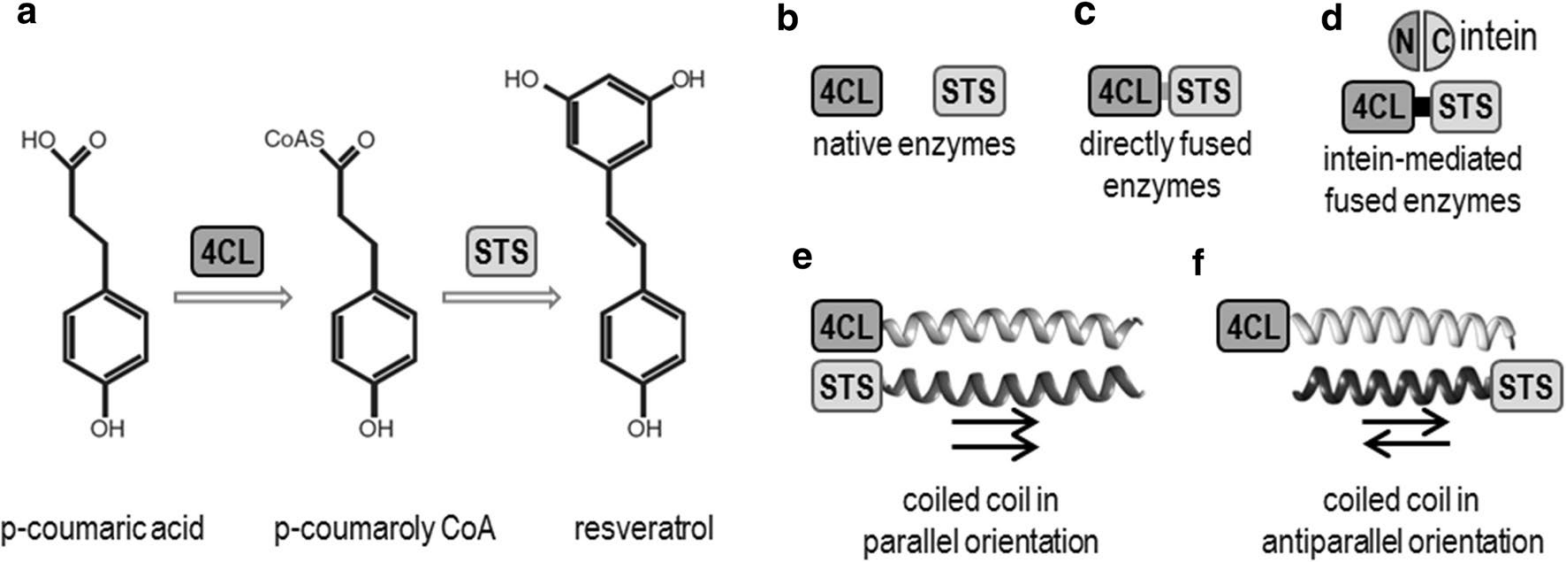
Schematic illustration of resveratrol biosynthesis and different strategies for the protein-based clustering of metabolic enzymes. **a** The enzymes 4-coumarate CoA ligase (4CL) and stilbene synthases (STS) are involved in resveratrol production from p-coumaric acid. **b** Native enzymes were used as a reference for resveratrol quantification. **c, d** Two strategies were based on the covalent fusion of the enzymes 4CL and STS: direct genetically encoded fusion (**c**) and intein-mediated fusion (**d**). **e, f** The interactions of designed coiled-coil-forming domains bring both enzymes into proximity. Coiled-coil dimers can have either a parallel (**e**) or an antiparallel (**f**) orientation

**Fig. 2:**
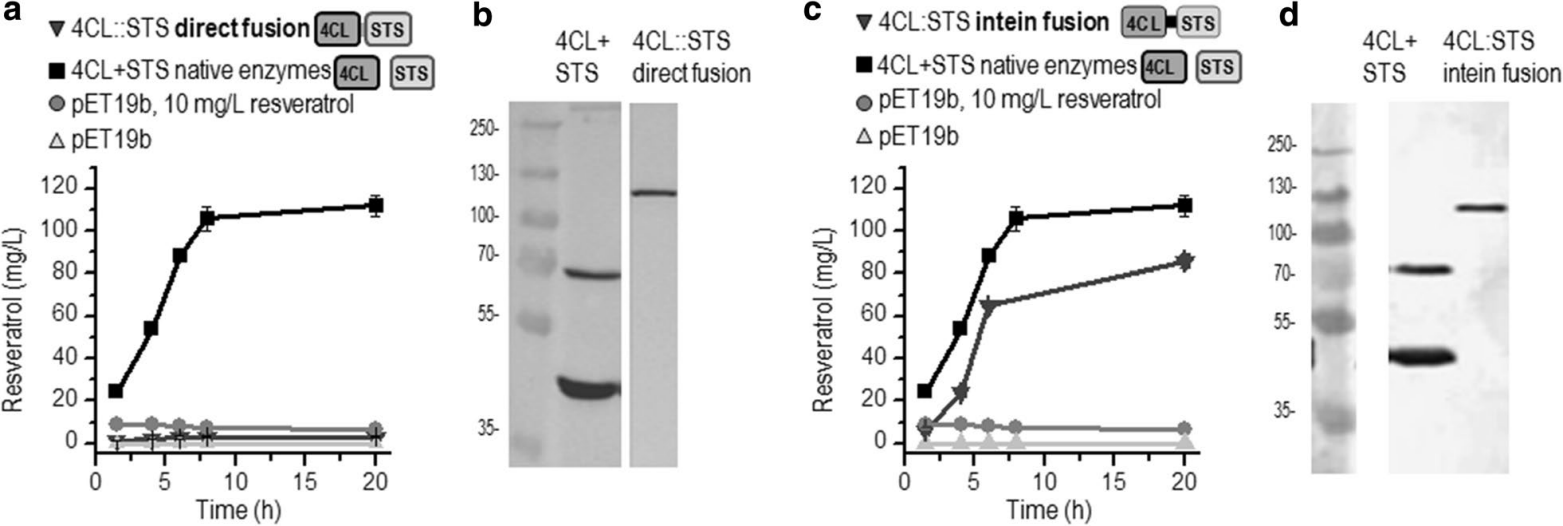
Comparison of the efficiency of covalently fused enzymes for resveratrol production. Resveratrol accumulation in *E. coli* strains expressing **a** the directly fused enzymes (4CL::STS) and **c** the enzymes covalently coupled via split intein fusion (4CL:STS). Expression of the directly fused (**b**) and intein-fused (**d**) enzymes was determined by WB. Native enzymes (4CL + STS) were used for reference. The data represent the average and standard deviation of the representative fermentations from three independent experiments with similar results

We evaluated several protein clustering strategies for the two-step production of resveratrol from p-coumaric acid (Fig. 1). As an initial attempt to generate macromolecular complexes in the biosynthetic pathway of resveratrol in *E. coli*, the enzymes 4-coumarate-CoA ligase (4CL) and stilbene synthase (STS) (Fig. 1a, b) were covalently fused via a genetically encoded construct using a 10-amino acid linker (Fig. 1c, sequence in Additional file 1: Table S1). Surprisingly, the analysis of resveratrol production in *E. coli* cells transformed with pET19b-(4CL-STS) direct fusion and, supplemented with p-coumaric acid as a precursor, resulted in no detectable resveratrol accumulation (Fig. 2a) even though the Western blot analysis clearly showed the presence of a protein with a molecular size of 112 kDa, corresponding with the expected size of the fusion protein (Fig. 2b).

The second strategy for expressing covalently fused enzymes involved the use of split inteins. In this study, the N-segment of the NpuDnaE split intein was fused to the C-terminus of the 4CL, and the C-segment of the NpuDnaE split intein was fused to the N-terminus of the STS. This should result in covalently fused 4CL and STS with a 10-amino acid linker (Fig. 1d, sequence in Additional file 1: Table S1), exactly as designed for the direct-fusion approach. This strategy successfully bypassed the potential folding problem inherent in fused multi-domain proteins, as confirmed by an analysis of resveratrol production and protein expression (Fig. 2c, d). The expression of protein (112 kDa) corresponding to the expected size of the covalent split intein-mediated fusion was confirmed by Western blot (Fig. 2d), indicating virtually complete intein fusion. Within 20 h of the induction, the resveratrol production reached 83 mg/L (Fig. 2c). Nevertheless, the resveratrol production level was 25% lower when compared with the reference strain, where enzymes 4CL and STS were expressed separately but simultaneously. In comparison with the separately expressed enzymes, however, the band intensity of intein fusion was weaker (Fig. 2d), suggesting reduced protein expression or decreased stability, which may explain the lower level of resveratrol accumulation.

### Clustering of biosynthetic enzymes through coiled-coil-forming domain interaction

Another strategy was clustering enzymes through noncovalent interactions of small protein domains. Here, our aim was to test coiled-coil-forming domains for the improvement of biosynthetic reactions. To organize two enzymes from the resveratrol biosynthetic pathway into clusters, coiled-coil-forming domains were genetically fused to the 4CL and STS at their C-termini. The 4CL and STS were each fused to a coiled-coil-forming domain with the validated propensity for the formation of a heterodimeric coiled coil within its designed pairwise partner. The formation of a coiled-coil dimer brings the enzymes into proximity, where the distance between the enzyme domains can be varied by the parallel or antiparallel orientation between the coiled-coil partners (Fig. 1e, f).

**Fig. 3:**
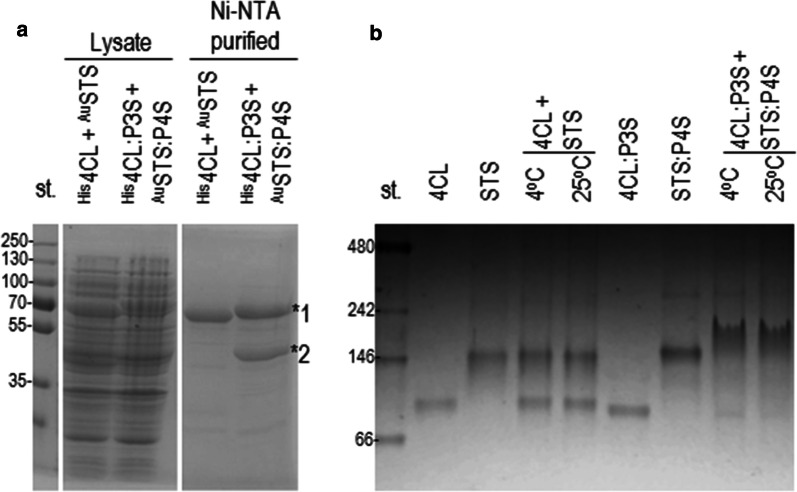
NiNTA-isolated coiled-coil-forming domains fused with enzymes interact and form clusters in vitro. **a** The SDS PAGE of the NiNTA-isolated proteins demonstrates the interaction between P3S and P4S from fusion proteins ^His^4CL:P3S (*1) and ^AU^STS:P4S (*2). **b** Native PAGE analysis revealed that in vitro clusters of 4CL and STS are formed at two different temperatures only on the fusion of enzymes with the coiled-coil-forming segments P3S and P4S

First we checked a formation of the enzyme cluster through coiled-coil-forming domain interaction using a parallel heterodimer P3S/P4S from our set of coiled coils (sequences in Additional file 1: Table S2). After the co-expression of ^His^4CL:P3S and ^AU^STS:P4S proteins in bacteria, the isolation was performed on the Ni–NTA affinity column, which selectively binds His-tagged proteins. We assumed that ^AU^STS:P4S protein co-isolates with the ^His^4CL:P3S protein if they form a P3S/P4S dimer based on the interaction of both coiled-coil-forming domains. This was indeed confirmed by SDS PAGE (Fig. 3a). In contrast, the two enzymes were not co-isolated in the coiled-coil-free enzymes ^His^4CL and ^AU^STS although the SDS PAGE analysis of the bacterial lysates revealed their expression (Fig. 3a). Next, the interaction between the coiled-coil-forming domains was also demonstrated by a native PAGE (Fig. 3b). The mixtures of the Ni–NTA isolated proteins ^His^4CL and ^His^STS or ^His^4CL:P3S and ^His^STS:P4S were incubated at 4 °C and 25 °C. A native PAGE of a mixture containing 4CL:P3S and STS:P4S revealed a band of ~ 200 kDa (Fig. 3b), which corresponds to the complex 4CL:P3S/STS:P4S, confirming the interaction between coiled-coil-forming domains P3S and P4S. However, we could not detect any enzyme complex in a control mixture consisting of the enzymes 4CL and STS without fused coiled-coil forming domains (Fig. 3b).

### Coiled-coil fusions for in vivo bacterial resveratrol production

We have further analyzed resveratrol production using coiled-coil-based enzyme clustering. The enzymes 4CL and STS were fused to coiled-coil-forming domains with different dimer stabilities and orientations (Fig. 1e, f). Several parallel coiled coils (Fig. 1e) with different levels of stability and helical propensity were therefore selected from our set: from the most stable pair, P5SHb/P6SHb, followed by P3S/P4S, to the less stable pairs, P7SHb/P8SHb and P7S/P8S, respectively (sequences in Additional file 1: Table S2) (Drobnak et al. 2017). In addition, an antiparallel coiled-coil-forming pair, P3S-AP4S (Fig. 1f), was tested to check whether the close proximity of the enzymes in parallel variants (Fig. 1e) might be hindered sterically and what the effect of the distance between the enzymes is.

**Fig. 4:**
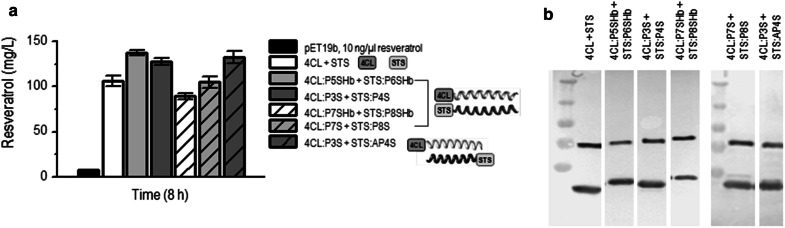
The clustering of biosynthetic enzymes via coiled-coil-forming domains improves resveratrol production in *E. coli*. **a** Enhancement of resveratrol biosynthesis in *E. coli* after 8 h, depending on the type of coiled-coil-forming domain fused to the enzymes. Simultaneously expressed native enzymes (4CL + STS) served as a reference. **b** The expression of enzymes coupled via coiled-coil-forming domain fusion was determined by WB. The same protein expression in three representative fermentations is shown in Additional file 1: Fig. S2

The enzymes fused to the most stable coiled coil, P5SHb-P6SHb, resulted in the highest resveratrol titer (145 mg/L) after 8 h of cultivation (Fig. 4a and Additional file 1: Fig. S2a). This was followed by a fusion protein with a slightly less stable coiled coil, P3S-P4S (125 mg/L). No significant differences in resveratrol accumulation were observed when using the parallel (P3S-P4S) or antiparallel (P3S-AP4S) coiled-coil variants. In all three coiled-coil fusions described above, resveratrol accumulation was significantly improved in comparison with the control strain with native enzymes (110 mg/L). Two of the selected coiled-coil pairs with low thermal stability, P7SHb-P8SHb and P7S-P8S, resulted in slightly lower titers than the control strain. However, all the fused proteins were expressed at the same level as determined by Western blot (Fig. 4b and Additional file 1: Fig. S2b). Additional file 1: Figure S2 shows the time course for resveratrol production and the expression of proteins when different enzyme-coiled-coil pairs were expressed in *E. coli*. We conclude, therefore, that a higher stability of a coiled-coil pair in enzyme fusion leads to a higher level of resveratrol production for around 30% in comparison to native enzymes.

### Coiled-coil fusions for the mevalonate production in yeast

**Fig. 5:**
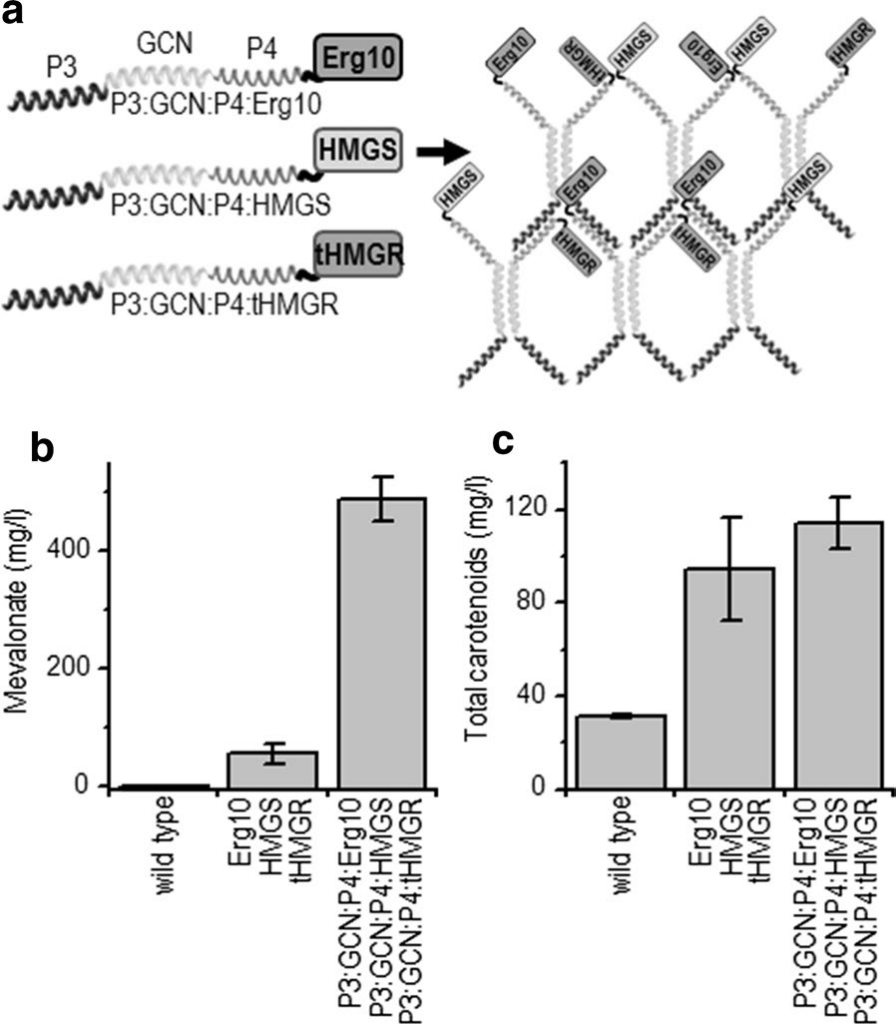
A coiled-coil dimer-based scaffold linked to mevalonate biosynthesis enzymes improves mevalonate accumulation in *S. cerevisiae*. **a** Scheme for the designed coiled-coil-based polypeptide scaffold. Extracellular mevalonate production (**b**) and total carotenoid accumulation (**c**) in carotenoid-producing yeast cells. The wild type is strain CAR-0002. CAR-0002 transformants express non-fused mevalonate pathway enzymes (Mev: ERG10, HMGS, or tHMGR) or coiled-coil scaffolded proteins (P3:GCN:P4-Mev). The data represent the average and standard deviation for mevalonate or the total carotenoid measurements for three independent transformants grown in duplicate

Finally, the coiled-coil scaffold-fused enzymes were introduced into *S. cerevisiae*, modifying three enzymes required for the production of mevalonate: ERG10, HMGS, and tHMGR. The scaffold-forming polypeptide was composed of three concatenated coiled-coil-forming domains: a homodimer-forming GCN, a heterodimer-forming P3, and a heterodimer-forming P4, which were designed to form a coiled-coil-mediated network. The fusion polypeptide P3:GCN:P4 was N-terminally fused to each of the mevalonate biosynthetic enzymes (Fig. 5a). The expression of enzymes with or without scaffold-forming domains was under the control of constitutive promoters. The yeast fermentations demonstrated an 8.8- fold increase in mevalonate accumulation after 72 h in strains where the mevalonate enzymes were clustered through scaffold formation in comparison to strains expressing non-scaffolded enzymes (Fig. 5b). Mass spectrometric analysis of the enzymes showed equal protein expression (data not shown), suggesting that the increased yield occurred due to scaffolding rather than due to the increased number of enzymes. Although the overexpression of non-scaffolded mevalonate pathway enzymes resulted in the increased production of carotenoids, a similar significant increase as that seen for mevalonate was not observed with scaffolded enzymes (Fig. 5c).

## Discussion

The clustering of biosynthetic enzymes can be achieved through different strategies, including direct fusion (Zhang et al. 2006), intein-mediated fusion (Selgrade et al. 2013), and fusion with natural or designed interaction domains (Conrado et al. 2012; Diener et al. 2016; Han et al. 2017; Yang et al. 2017). However, protein-based scaffolds as enzyme clustering principle have, since they were reported, demonstrated use in different biosynthetic reactions. Most of the results demonstrated based on protein-peptide domain interactions (e.g., SH3, PDZ, GBD), where the binding domains were relatively large and were based on different protein folds, may have different physicochemical properties and may interfere with natural ligands, particularly in eukaryotic cells. Coiled-coil dimers, alternatively, are available in several orthogonal sets (Gradišar and Jerala 2011; Reinke et al. 2013), have similar properties, and are relatively small in comparison to the enzymes. The use of a coiled-coil single leucine zipper pair has been previously reported to increase the production of 1-butanol (Han et al. 2017); however, here we report on the use of a set of orthogonal coiled-coil pairs (Drobnak et al. 2017), which can be tuned for stability and are designed de novo to avoid interference with natural coiled coils.

We tested several strategies of enzyme clustering, and as a model we chose metabolic enzymes 4CL and STS from a resveratrol metabolic pathway. First, we prepared a direct fusion of both enzymes. The fusion protein was expressed, but the enzyme activity was not detected. Although direct fusion has been reported to generate functional fused proteins that produce resveratrol in yeast (Zhang et al. 2006), the misfolding of the fused enzymes either due to the steric hindrance during protein folding or caused by the lack of required folding chaperones inside the *E. coli* cells could be responsible for the failure of this strategy.

We assumed that the strategy using split inteins could lead to a functional enzyme fusion. Inteins are self-splicing elements that can generate fused polypeptide chains from a pair of separately translated polypeptide chains with virtually no remaining scars (Shah and Muir 2011; Aranko et al. 2014). The main advantage of this approach, in comparison with direct protein fusion, is the independent folding of each individual enzyme as they are fused to a relatively small split intein domain, thereby eliminating the mutual folding interference between the polypeptide chains. Each split intein segment is fused to an individual enzyme domain and is coded, transcribed, and translated separately. After translation, pairs of proteins with separate split domains interact and can form a covalent bond in which virtually all the intein amino acid sequence is excised from the final fusion protein. Our results confirmed the expression of intein-based fusion, but the production of resveratrol was 25% lower compared to the reference separately but simultaneously expressed enzymes 4CL and STS.

An alternative strategy for bringing biosynthetic enzymes into proximity involves inducing noncovalent clustering mediated by small protein interaction domains. By adding short protein interacting domains at the N- or C-terminus of both enzyme partners, stable noncovalent protein clusters can be expressed. To combine more than a single pair of catalytic domains selectively, several orthogonal interaction partners are needed, which could be provided by an orthogonal set of designed coiled-coil dimers. Previously, we designed such a toolbox of several orthogonal heterodimeric coiled coils (Gradišar and Jerala 2011; Drobnak et al. 2017), reporting on their application as modular building blocks for in vitro and in vivo protein-cage formation (Gradišar et al. 2013; Ljubetič et al. 2017).

First, we proved a formation of enzyme clusters through coiled-coil-forming domain interaction. Using fusions between enzymes and coiled-coil-forming domains, 4CL:P3S and STS:P4S, respectively, we showed that complex 4CL:P3S/STS:P4S was formed. For the production of resveratrol, we tested several parallel coiled coils with different levels of stability and helical propensity. For the most stable pairs, P5SHb/P6SHb and P3S/P4S, the production of resveratrol increased up to 30% compared to the reference. Further, the resveratrol production was comparable for enzyme fusions with parallel coiled-coil P3S/P4S and its antiparallel variant P3S/AP4S. This result suggests that at least in the case of enzymes on the resveratrol pathway, small differences in enzyme proximity due to the coiled-coil orientation (~ 4 nm) do not significantly influence the overall biosynthetic yield. Overall, the results indicate that coiled-coil-based dimers increase the biosynthetic product yield for resveratrol and that the improvement is correlated with coiled-coil stability. In the experiments, the amounts of free and coiled-coil-fused enzymes did not vary significantly. It is also possible that coiled-coil fusion may increase enzyme stability or proteolytic resistance, which could also contribute to increased yields.

Finally, to investigate whether the coiled-coil protein fusion platform could be extended to other organisms and could be used for the biosynthesis of other multi-catalytic-step biosynthetic products, the coiled-coil scaffold-fused enzymes were introduced into *S. cerevisiae.* We prepared enzyme fusions with domains composed of three coiled-coil-forming segments that can form not only dimers but larger scaffolds. The constructs were transformed into yeast strain CAR0002, which contains the carotenoid biosynthesis pathway from *Xanthoplyllomyces dendrorhous* (Zhang et al. 2006). In this pathway, mevalonate serves as a precursor for carotenoid production, thus measuring the carotenoid production serves as an additional reporter of mevalonate production. After 72 h, the production of mevalonate was remarkably higher (8.8-fold induction) compared to the control (non-scaffolded enzymes). The carotenoid amount had also increased, but the difference was not significant. Larger increases may be observed when flux through the carotenoid pathway is improved, such as by the introduction of additional copies of carotenogenic genes (Verwaal et al. 2007). In summary, these results demonstrate that coiled-coil-based enzyme clustering is also functional in yeast.

Although different biosynthetic pathways cannot be compared directly, our results show that the yield improvement was largest in the case of concatenated coiled-coil-forming domains. This is likely due to the potential to lead to the formation of larger clusters, which has been proposed to be required for an increased biosynthetic yield (Lee et al. 2012). In the future, the availability of our large set of designed orthogonal coiled-coil dimers/oligomers will enable the design of defined clusters for multi-component biosynthetic pathways.

## Supplementary information

**Additional file 1.** The additional information accompanies this paper at https://amb-express.springeropen.com/.

## Data Availability

Corresponding author could provide all the experimental data on valid request. The nucleotide sequences of the codon-optimized synthetic genes were deposited at the Addgene and NCBI GenBank databases. The accession numbers are listed in the section Materials and methods.

## Abbreviations

CC: Coiled coil
4CL: 4-coumaroyl-CoA ligase
STS: Stilbene synthase
Erg10: Acetyl-CoA acetyl transferase
HMGS: HMG-CoA synthase
tHMGR: Truncated HMG-CoA reductase
