# Supplementary material for: Metabolic enzyme clustering by coiled coils improves the biosynthesis of resveratrol and mevalonate
Source: AMB Express. 2020 May 24;10:97. doi: 10.1186/s13568-020-01031-5 (PMC7246283; doi:10.1186/s13568-020-01031-5)
Supplement: Supplementary file 1 — Additional file 1. The additional information accompanies this paper at https://amb-express.springeropen.com/. [file 13568_2020_1031_MOESM1_ESM.docx]

**Additional material**

**Metabolic enzyme clustering by coiled coils improves the biosynthesis of resveratrol and mevalonate**

Tina Fink^1^, Bojana Stevović^1^, René Verwaal^2^, Johannes A. Roubos^2^, Rok Gaber^1^, Mojca Benčina^1,3^, Roman Jerala^1,3,^* and Helena Gradišar^1,3,^*

^1^ Department of Synthetic Biology and Immunology, National Institute of Chemistry, Ljubljana, Slovenia

^2^ DSM Biotechnology Center, DSM, Delft, the Netherlands

^3^ EN-FIST Centre of Excellence, Ljubljana, Slovenia

*Corresponding authors at: Department of Synthetic Biology and Immunology, National Institute of Chemistry, Hajdrihova 19, SI-1000 Ljubljana, Slovenia

E-mail: [roman.jerala@ki.si](mailto:roman.jerala@ki.si) (R. Jerala); [helena.gradisar@ki.si](mailto:helena.gradisar@ki.si) (H. Gradišar)

**Additional Tables S1, S2**

**Additional Figures S1, S2**

**Table S1** Amino acid sequences of proteins. Amino acids in the linker between segments are underlined.

| ***Proteins*** | ***Amino acid sequences*** |
| --- | --- |
| **4CL** | MHHHHHHEKQSNNNNSDVIFRSKLPDIYIPNHLSLHDYIFQNISEFATKPCLINGPTGHVYTYSDVHVISRQIAANFHKLGVNQNDVVMLLLPNCPEFVLSFLAASFRGATATAANPFFTPAEIAKQAKASNTKLIITEARYVDKIKPLQNDDGVVIVCIDDNESVPIPEGCLRFTELTQSTTEASEVIDSVEISPDDVVALPYSSGTTGLPKGVMLTHKGLVTSVAQQVDGENPNLYFHSDDVILCVLPMFHIYALNSIMLCGLRVGAAILIMPKFEINLLLELIQRCKVTVAPMVPPIVLAIAKSSETEKYDLSSIRVVKSGAAPLGKELEDAVNAKFPNAKLGQGYGMTEAGPVLAMSLGFAKEPFPVKSGACGTVVRNAEMKIVDPDTGDSLSRNQPGEICIRGHQIMKGYLNNPAATAETIDKDGWLHTGDIGLIDDDDELFIVDRLKELIKYKGFQVAPAELEALLIGHPDITDVAVVAMKEEAAGEVPVAFVVKSKDSELSEDDVKQFVSKQVVFYKRINKVFFTESIPKAPSGKILRKDLRAKLANGL |
| **STS** | MHHHHHHASVEEFRNAQRAKGPATILAIGTATPDHCVYQSDYADYYFKVTKSEHMTALKKKFNRICDKSMIKKRYIHLTEEMLEEHPNIGAYMAPSLNIRQEIITAEVPKLGKEAALKALKEWGQPKSKITHLVFCTTSGVEMPGADYKLANLLGLEPSVRRVMLYHQGCYAGGTVLRTAKDLAENNAGARVLVVCSEITVVTFRGPSEDALDSLVGQALFGDGSAAVIVGSDPDISIERPLFQLVSAAQTFIPNSAGAIAGNLREVGLTFHLWPNVPTLISENVEKCLTQAFDPLGISDWNSLFWIAHPGGPAILDAVEAKLNLDKKKLEATRHVLSEYGNMSSACVLFILDEMRKKSLKGERATTGEGLDWGVLFGFGPGLTIETVVLHSIPMVTN |
| **4CL:STS**  **Direct fusion** | MHHHHHHSSGLVPRGSHMASMTGGQQMGRGSMAPQEQAVSQVMEKQSNNNNSDVIFRSKLPDIYIPNHLSLHDYIFQNISEFATKPCLINGPTGHVYTYSDVHVISRQIAANFHKLGVNQNDVVMLLLPNCPEFVLSFLAASFRGATATAANPFFTPAEIAKQAKASNTKLIITEARYVDKIKPLQNDDGVVIVCIDDNESVPIPEGCLRFTELTQSTTEASEVIDSVEISPDDVVALPYSSGTTGLPKGVMLTHKGLVTSVAQQVDGENPNLYFHSDDVILCVLPMFHIYALNSIMLCGLRVGAAILIMPKFEINLLLELIQRCKVTVAPMVPPIVLAIAKSSETEKYDLSSIRVVKSGAAPLGKELEDAVNAKFPNAKLGQGYGMTEAGPVLAMSLGFAKEPFPVKSGACGTVVRNAEMKIVDPDTGDSLSRNQPGEICIRGHQIMKGYLNNPAATAETIDKDGWLHTGDIGLIDDDDELFIVDRLKELIKYKGFQVAPAELEALLIGHPDITDVAVVAMKEEAAGEVPVAFVVKSKDSELSEDDVKQFVSKQVVFYKRINKVFFTESIPKAPSGKILRKDLRAKLANGLGSGMASVEEFRNAQRAKGPATILAIGTATPDHCVYQSDYADYYFKVTKSEHMTALKKKFNRICDKSMIKKRYIHLTEEMLEEHPNIGAYMAPSLNIRQEIITAEVPKLGKEAALKALKEWGQPKSKITHLVFCTTSGVEMPGADYKLANLLGLEPSVRRVMLYHQGCYAGGTVLRTAKDLAENNAGARVLVVCSEITVVTFRGPSEDALDSLVGQALFGDGSAAVIVGSDPDISIERPLFQLVSAAQTFIPNSAGAIAGNLREVGLTFHLWPNVPTLISENVEKCLTQAFDPLGISDWNSLFWIAHPGGPAILDAVEAKLNLDKKKLEATRHVLSEYGNMSSACVLFILDEMRKKSLKGERATTGEGLDWGVLFGFGPGLTIETVVLHSIPMVTN |
| **4CL:intN** | MHHHHHHEKQSNNNNSDVIFRSKLPDIYIPNHLSLHDYIFQNISEFATKPCLINGPTGHVYTYSDVHVISRQIAANFHKLGVNQNDVVMLLLPNCPEFVLSFLAASFRGATATAANPFFTPAEIAKQAKASNTKLIITEARYVDKIKPLQNDDGVVIVCIDDNESVPIPEGCLRFTELTQSTTEASEVIDSVEISPDDVVALPYSSGTTGLPKGVMLTHKGLVTSVAQQVDGENPNLYFHSDDVILCVLPMFHIYALNSIMLCGLRVGAAILIMPKFEINLLLELIQRCKVTVAPMVPPIVLAIAKSSETEKYDLSSIRVVKSGAAPLGKELEDAVNAKFPNAKLGQGYGMTEAGPVLAMSLGFAKEPFPVKSGACGTVVRNAEMKIVDPDTGDSLSRNQPGEICIRGHQIMKGYLNNPAATAETIDKDGWLHTGDIGLIDDDDELFIVDRLKELIKYKGFQVAPAELEALLIGHPDITDVAVVAMKEEAAGEVPVAFVVKSKDSELSEDDVKQFVSKQVVFYKRINKVFFTESIPKAPSGKILRKDLRAKLANGLGGSCLSYETEILTVEYGLLPIGKIVEKRIECTVYSVDNNGNIYTQPVAQWHDRGEQEVFEYCLEDGSLIRATKDHKFMTVDGQMLPIDEIFERELDLMRVDNLP |
| **intC:STS** | MHHHHHHIKIATRKYLGKQNVYDIGVERDHNFALKNGFIASNCFNGGASVEEFRNAQRAKGPATILAIGTATPDHCVYQSDYADYYFKVTKSEHMTALKKKFNRICDKSMIKKRYIHLTEEMLEEHPNIGAYMAPSLNIRQEIITAEVPKLGKEAALKALKEWGQPKSKITHLVFCTTSGVEMPGADYKLANLLGLEPSVRRVMLYHQGCYAGGTVLRTAKDLAENNAGARVLVVCSEITVVTFRGPSEDALDSLVGQALFGDGSAAVIVGSDPDISIERPLFQLVSAAQTFIPNSAGAIAGNLREVGLTFHLWPNVPTLISENVEKCLTQAFDPLGISDWNSLFWIAHPGGPAILDAVEAKLNLDKKKLEATRHVLSEYGNMSSACVLFILDEMRKKSLKGERATTGEGLDWGVLFGFGPGLTIETVVLHSIPMVTN |
| **4CL-P3S** | MHHHHHHHHHHSSGHIDDDDKHMAPQEQAVSQVMEKQSNNNNSDVIFRSKLPDIYIPNHLSLHDYIFQNISEFATKPCLINGPTGHVYTYSDVHVISRQIAANFHKLGVNQNDVVMLLLPNCPEFVLSFLAASFRGATATAANPFFTPAEIAKQAKASNTKLIITEARYVDKIKPLQNDDGVVIVCIDDNESVPIPEGCLRFTELTQSTTEASEVIDSVEISPDDVVALPYSSGTTGLPKGVMLTHKGLVTSVAQQVDGENPNLYFHSDDVILCVLPMFHIYALNSIMLCGLRVGAAILIMPKFEINLLLELIQRCKVTVAPMVPPIVLAIAKSSETEKYDLSSIRVVKSGAAPLGKELEDAVNAKFPNAKLGQGYGMTEAGPVLAMSLGFAKEPFPVKSGACGTVVRNAEMKIVDPDTGDSLSRNQPGEICIRGHQIMKGYLNNPAATAETIDKDGWLHTGDIGLIDDDDELFIVDRLKELIKYKGFQVAPAELEALLIGHPDITDVAVVAMKEEAAGEVPVAFVVKSKDSELSEDDVKQFVSKQVVFYKRINKVFFTESIPKAPSGKILRKDLRAKLANGLGGSGSSPEDEIQQLEEEISQLEQKNSELKEKNQELKYGY |
|  |  |
| **STS-P4S** | MHHHHHHHHHHSSGHIDDDDKHMASVEEFRNAQRAKGPATILAIGTATPDHCVYQSDYADYYFKVTKSEHMTALKKKFNRICDKSMIKKRYIHLTEEMLEEHPNIGAYMAPSLNIRQEIITAEVPKLGKEAALKALKEWGQPKSKITHLVFCTTSGVEMPGADYKLANLLGLEPSVRRVMLYHQGCYAGGTVLRTAKDLAENNAGARVLVVCSEITVVTFRGPSEDALDSLVGQALFGDGSAAVIVGSDPDISIERPLFQLVSAAQTFIPNSAGAIAGNLREVGLTFHLWPNVPTLISENVEKCLTQAFDPLGISDWNSLFWIAHPGGPAILDAVEAKLNLDKKKLEATRHVLSEYGNMSSACVLFILDEMRKKSLKGERATTGEGLDWGVLFGFGPGLTIETVVLHSIPMVTNGSGSSPEDKISQLKEKIQQLKQENQQLEEENSQLEYG |
| **4CL-P5SHb** | MHHHHHHHHHHSSGHIDDDDKHMAPQEQAVSQVMEKQSNNNNSDVIFRSKLPDIYIPNHLSLHDYIFQNISEFATKPCLINGPTGHVYTYSDVHVISRQIAANFHKLGVNQNDVVMLLLPNCPEFVLSFLAASFRGATATAANPFFTPAEIAKQAKASNTKLIITEARYVDKIKPLQNDDGVVIVCIDDNESVPIPEGCLRFTELTQSTTEASEVIDSVEISPDDVVALPYSSGTTGLPKGVMLTHKGLVTSVAQQVDGENPNLYFHSDDVILCVLPMFHIYALNSIMLCGLRVGAAILIMPKFEINLLLELIQRCKVTVAPMVPPIVLAIAKSSETEKYDLSSIRVVKSGAAPLGKELEDAVNAKFPNAKLGQGYGMTEAGPVLAMSLGFAKEPFPVKSGACGTVVRNAEMKIVDPDTGDSLSRNQPGEICIRGHQIMKGYLNNPAATAETIDKDGWLHTGDIGLIDDDDELFIVDRLKELIKYKGFQVAPAELEALLIGHPDITDVAVVAMKEEAAGEVPVAFVVKSKDSELSEDDVKQFVSKQVVFYKRINKVFFTESIPKAPSGKILRKDLRAKLANGLGGSGSPEDENRELEEKIRELKEKNEELKREIKYLEE |
| **STS-P6SHb** | MHHHHHHHHHHSSGHIDDDDKHMASVEEFRNAQRAKGPATILAIGTATPDHCVYQSDYADYYFKVTKSEHMTALKKKFNRICDKSMIKKRYIHLTEEMLEEHPNIGAYMAPSLNIRQEIITAEVPKLGKEAALKALKEWGQPKSKITHLVFCTTSGVEMPGADYKLANLLGLEPSVRRVMLYHQGCYAGGTVLRTAKDLAENNAGARVLVVCSEITVVTFRGPSEDALDSLVGQALFGDGSAAVIVGSDPDISIERPLFQLVSAAQTFIPNSAGAIAGNLREVGLTFHLWPNVPTLISENVEKCLTQAFDPLGISDWNSLFWIAHPGGPAILDAVEAKLNLDKKKLEATRHVLSEYGNMSSACVLFILDEMRKKSLKGERATTGEGLDWGVLFGFGPGLTIETVVLHSIPMVTNGSGSPEDKNEELKREIERLEEENRELERKIEYLKR |
| **4CL-P7S** | MHHHHHHHHHHSSGHIDDDDKHMAPQEQAVSQVMEKQSNNNNSDVIFRSKLPDIYIPNHLSLHDYIFQNISEFATKPCLINGPTGHVYTYSDVHVISRQIAANFHKLGVNQNDVVMLLLPNCPEFVLSFLAASFRGATATAANPFFTPAEIAKQAKASNTKLIITEARYVDKIKPLQNDDGVVIVCIDDNESVPIPEGCLRFTELTQSTTEASEVIDSVEISPDDVVALPYSSGTTGLPKGVMLTHKGLVTSVAQQVDGENPNLYFHSDDVILCVLPMFHIYALNSIMLCGLRVGAAILIMPKFEINLLLELIQRCKVTVAPMVPPIVLAIAKSSETEKYDLSSIRVVKSGAAPLGKELEDAVNAKFPNAKLGQGYGMTEAGPVLAMSLGFAKEPFPVKSGACGTVVRNAEMKIVDPDTGDSLSRNQPGEICIRGHQIMKGYLNNPAATAETIDKDGWLHTGDIGLIDDDDELFIVDRLKELIKYKGFQVAPAELEALLIGHPDITDVAVVAMKEEAAGEVPVAFVVKSKDSELSEDDVKQFVSKQVVFYKRINKVFFTESIPKAPSGKILRKDLRAKLANGLGGSGSPEDEIQSLEEKNSQLKQEISQLEEKNQQLKYG |
| **STS-P8S** | MHHHHHHHHHHSSGHIDDDDKHMASVEEFRNAQRAKGPATILAIGTATPDHCVYQSDYADYYFKVTKSEHMTALKKKFNRICDKSMIKKRYIHLTEEMLEEHPNIGAYMAPSLNIRQEIITAEVPKLGKEAALKALKEWGQPKSKITHLVFCTTSGVEMPGADYKLANLLGLEPSVRRVMLYHQGCYAGGTVLRTAKDLAENNAGARVLVVCSEITVVTFRGPSEDALDSLVGQALFGDGSAAVIVGSDPDISIERPLFQLVSAAQTFIPNSAGAIAGNLREVGLTFHLWPNVPTLISENVEKCLTQAFDPLGISDWNSLFWIAHPGGPAILDAVEAKLNLDKKKLEATRHVLSEYGNMSSACVLFILDEMRKKSLKGERATTGEGLDWGVLFGFGPGLTIETVVLHSIPMVTNGSGSPEDKISQLKEENQQLEQKIQQLKEENSQLEYG |
| **4CL-P7SHb** | MHHHHHHHHHHSSGHIDDDDKHMAPQEQAVSQVMEKQSNNNNSDVIFRSKLPDIYIPNHLSLHDYIFQNISEFATKPCLINGPTGHVYTYSDVHVISRQIAANFHKLGVNQNDVVMLLLPNCPEFVLSFLAASFRGATATAANPFFTPAEIAKQAKASNTKLIITEARYVDKIKPLQNDDGVVIVCIDDNESVPIPEGCLRFTELTQSTTEASEVIDSVEISPDDVVALPYSSGTTGLPKGVMLTHKGLVTSVAQQVDGENPNLYFHSDDVILCVLPMFHIYALNSIMLCGLRVGAAILIMPKFEINLLLELIQRCKVTVAPMVPPIVLAIAKSSETEKYDLSSIRVVKSGAAPLGKELEDAVNAKFPNAKLGQGYGMTEAGPVLAMSLGFAKEPFPVKSGACGTVVRNAEMKIVDPDTGDSLSRNQPGEICIRGHQIMKGYLNNPAATAETIDKDGWLHTGDIGLIDDDDELFIVDRLKELIKYKGFQVAPAELEALLIGHPDITDVAVVAMKEEAAGEVPVAFVVKSKDSELSEDDVKQFVSKQVVFYKRINKVFFTESIPKAPSGKILRKDLRAKLANGLGGSGSPEDEIRELEEKNEELKREIRELEEKNEYLKR |
| **STS-P8SHb** | MHHHHHHHHHHSSGHIDDDDKHMASVEEFRNAQRAKGPATILAIGTATPDHCVYQSDYADYYFKVTKSEHMTALKKKFNRICDKSMIKKRYIHLTEEMLEEHPNIGAYMAPSLNIRQEIITAEVPKLGKEAALKALKEWGQPKSKITHLVFCTTSGVEMPGADYKLANLLGLEPSVRRVMLYHQGCYAGGTVLRTAKDLAENNAGARVLVVCSEITVVTFRGPSEDALDSLVGQALFGDGSAAVIVGSDPDISIERPLFQLVSAAQTFIPNSAGAIAGNLREVGLTFHLWPNVPTLISENVEKCLTQAFDPLGISDWNSLFWIAHPGGPAILDAVEAKLNLDKKKLEATRHVLSEYGNMSSACVLFILDEMRKKSLKGERATTGEGLDWGVLFGFGPGLTIETVVLHSIPMVTNGSGSPEDKIEELKRENRELERKIRELKRENEYLER |
| **STS-AP4S** | MHHHHHHHHHHSSGHIDDDDKHMASVEEFRNAQRAKGPATILAIGTATPDHCVYQSDYADYYFKVTKSEHMTALKKKFNRICDKSMIKKRYIHLTEEMLEEHPNIGAYMAPSLNIRQEIITAEVPKLGKEAALKALKEWGQPKSKITHLVFCTTSGVEMPGADYKLANLLGLEPSVRRVMLYHQGCYAGGTVLRTAKDLAENNAGARVLVVCSEITVVTFRGPSEDALDSLVGQALFGDGSAAVIVGSDPDISIERPLFQLVSAAQTFIPNSAGAIAGNLREVGLTFHLWPNVPTLISENVEKCLTQAFDPLGISDWNSLFWIAHPGGPAILDAVEAKLNLDKKKLEATRHVLSEYGNMSSACVLFILDEMRKKSLKGERATTGEGLDWGVLFGFGPGLTIETVVLHSIPMVTNGSGSSPEDELQSNEEELQQNEQKLQQIKQKLQSIKYG |
| **Erg10** | MSQNVYIVSTARTPIGSFQGSLSSKTAVELGAVALKGALAKVPELDASKDFDEIIFGNVLSANLGQAPARQVALAAGLSNHIVASTVNKVCASAMKAIILGAQSIKCGNADVVVAGGCESMTNAPYYMPAARAGAKFGQTVLVDGVERDGLNDAYDGLAMGVHAEKCARDWDITREQQDNFAIESYQKSQKSQKEGKFDNEIVPVTIKGFRGKPDTQVTKDEEPARLHVEKLRSARTVFQKENGTVTAANASPINDGAAAVILVSEKVLKEKNLKPLAIIKGWGEAAHQPADFTWAPSLAVPKALKHAGIEDINSVDYFEFNEAFSVVGLVNTKILKLDPSKVNVYGGAVALGHPLGCSGARVVVTLLSILQQEGGKIGVAAICNGGGGASSIVIEKI |
| **HMGS** | MKLSTKLCWCGIKGRLRPQKQQQLHNTNLQMTELKKQKTAEQKTRPQNVGIKGIQIYIPTQCVNQSELEKFDGVSQGKYTIGLGQTNMSFVNDREDIYSMSLTVLSKLIKSYNIDTNKIGRLEVGTETLIDKSKSVKSVLMQLFGENTDVEGIDTLNACYGGTNALFNSLNWIESNAWDGRDAIVVCGDIAIYDKGAARPTGGAGTVAMWIGPDAPIVFDSVRASYMEHAYDFYKPDFTSEYPYVDGHFSLTCYVKALDQVYKSYSKKAISKGLVSDPAGSDALNVLKYFDYNVFHVPTCKLVTKSYGRLLYNDFRANPQLFPEVDAELATRDYDESLTDKNIEKTFVNVAKPFHKERVAQSLIVPTNTGNMYTASVYAAFASLLNYVGSDDLQGKRVGLFSYGSGLAASLYSCKIVGDVQHIIKELDITNKLAKRITETPKDYEAAIELRENAHLKKNFKPQGSIEHLQSGVYYLTNIDDKFRRSYDVKK |
| **tHMGR** | MDQLVKTEVTKKSFTAPVQKASTPVLTNKTVISGSKVKSLSSAQSSSSGPSSSSEEDDSRDIESLDKKIRPLEELEALLSSGNTKQLKNKEVAALVIHGKLPLYALEKKLGDTTRAVAVRRKALSILAEAPVLASDRLPYKNYDYDRVFGACCENVIGYMPLPVGVIGPLVIDGTSYHIPMATTEGCLVASAMRGCKAINAGGGATTVLTKDGMTRGPVVRFPTLKRSGACKIWLDSEEGQNAIKKAFNSTSRFARLQHIQTCLAGDLLFMRFRTTTGDAMGMNMISKGVEYSLKQMVEEYGWEDMEVVSVSGNYCTDKKPAAINWIEGRGKSVVAEATIPGDVVRKVLKSDVSALVELNIAKNLVGSAMAGSVGGFNAHAANLVTAVFLALGQDPAQNVESSNCITLMKEVDGDLRISVSMPSIEVGTIGGGTVLEPQGAMLDLLGVRGPHATAPGTNARQLARIVACAVLAGELSLCAALAAGHLVQSHMTHNRKPAEPTKPNNLDATDINRLKDGSVTCIKS |
| **P3:GCN:P4 (CC)** | MGSSPEDEIQQLEEEIAQLEQKNAALKEKNQALKYGSGRMKQLEDKIEELLSKIYHLENEIARLKKLIGERSGSPEDKIAQLKQKIQALKQENQQLEEENAALEYG |
| **CC-Erg10** | MGSSPEDEIQQLEEEIAQLEQKNAALKEKNQALKYGSGRMKQLEDKIEELLSKIYHLENEIARLKKLIGERSGSPEDKIAQLKQKIQALKQENQQLEEENAALEYGGSGGSGSGGSSQNVYIVSTARTPIGSFQGSLSSKTAVELGAVALKGALAKVPELDASKDFDEIIFGNVLSANLGQAPARQVALAAGLSNHIVASTVNKVCASAMKAIILGAQSIKCGNADVVVAGGCESMTNAPYYMPAARAGAKFGQTVLVDGVERDGLNDAYDGLAMGVHAEKCARDWDITREQQDNFAIESYQKSQKSQKEGKFDNEIVPVTIKGFRGKPDTQVTKDEEPARLHVEKLRSARTVFQKENGTVTAANASPINDGAAAVILVSEKVLKEKNLKPLAIIKGWGEAAHQPADFTWAPSLAVPKALKHAGIEDINSVDYFEFNEAFSVVGLVNTKILKLDPSKVNVYGGAVALGHPLGCSGARVVVTLLSILQQEGGKIGVAAICNGGGGASSIVIEKI |
| **CC-HMGS** | MGSSPEDEIQQLEEEIAQLEQKNAALKEKNQALKYGSGRMKQLEDKIEELLSKIYHLENEIARLKKLIGERSGSPEDKIAQLKQKIQALKQENQQLEEENAALEYGGSGGSGSGGSKLSTKLCWCGIKGRLRPQKQQQLHNTNLQMTELKKQKTAEQKTRPQNVGIKGIQIYIPTQCVNQSELEKFDGVSQGKYTIGLGQTNMSFVNDREDIYSMSLTVLSKLIKSYNIDTNKIGRLEVGTETLIDKSKSVKSVLMQLFGENTDVEGIDTLNACYGGTNALFNSLNWIESNAWDGRDAIVVCGDIAIYDKGAARPTGGAGTVAMWIGPDAPIVFDSVRASYMEHAYDFYKPDFTSEYPYVDGHFSLTCYVKALDQVYKSYSKKAISKGLVSDPAGSDALNVLKYFDYNVFHVPTCKLVTKSYGRLLYNDFRANPQLFPEVDAELATRDYDESLTDKNIEKTFVNVAKPFHKERVAQSLIVPTNTGNMYTASVYAAFASLLNYVGSDDLQGKRVGLFSYGSGLAASLYSCKIVGDVQHIIKELDITNKLAKRITETPKDYEAAIELRENAHLKKNFKPQGSIEHLQSGVYYLTNIDDKFRRSYDVKK |
| **CC-tHMGR** | MGSSPEDEIQQLEEEIAQLEQKNAALKEKNQALKYGSGRMKQLEDKIEELLSKIYHLENEIARLKKLIGERSGSPEDKIAQLKQKIQALKQENQQLEEENAALEYGGSGGSGSGGSDQLVKTEVTKKSFTAPVQKASTPVLTNKTVISGSKVKSLSSAQSSSSGPSSSSEEDDSRDIESLDKKIRPLEELEALLSSGNTKQLKNKEVAALVIHGKLPLYALEKKLGDTTRAVAVRRKALSILAEAPVLASDRLPYKNYDYDRVFGACCENVIGYMPLPVGVIGPLVIDGTSYHIPMATTEGCLVASAMRGCKAINAGGGATTVLTKDGMTRGPVVRFPTLKRSGACKIWLDSEEGQNAIKKAFNSTSRFARLQHIQTCLAGDLLFMRFRTTTGDAMGMNMISKGVEYSLKQMVEEYGWEDMEVVSVSGNYCTDKKPAAINWIEGRGKSVVAEATIPGDVVRKVLKSDVSALVELNIAKNLVGSAMAGSVGGFNAHAANLVTAVFLALGQDPAQNVESSNCITLMKEVDGDLRISVSMPSIEVGTIGGGTVLEPQGAMLDLLGVRGPHATAPGTNARQLARIVACAVLAGELSLCAALAAGHLVQSHMTHNRKPAEPTKPNNLDATDINRLKDGSVTCIKS |

**Table S2** List of coiled-coil pairs used in this study: their amino acid sequences, orientations and thermal stabilities (Gradišar and Jerala 2011; Drobnak et al. 2017).

| ***Coiled-coil pairs*** | ***Amino acid sequences*** | ***Orientation*** | ***Thermal Stabilities*** |
| --- | --- | --- | --- |
| **P3S** | SPEDEIQQLEEEISQLEQKNSELKEKNQELKYGY | parallel | 38 °C |
| **P4S** | SPEDKISQLKEKIQQLKQENQQLEEENSQLEYG | heterodimer |  |
| **P5SHb** | SPEDENRELEEKIRELKEKNEELKREIKYLEE | parallel | 53 °C |
| **P6SHb** | SPEDKNEELKREIERLEEENRELERKIEYLKR | heterodimer |  |
| **P7S** | SPEDEIQSLEEKNSQLKQEISQLEEKNQQLKYG | parallel | < 15 °C |
| **P8S** | SPEDKISQLKEENQQLEQKIQQLKEENSQLEYG | heterodimer |  |
| **P7SHb** | SPEDEIRELEEKNEELKREIRELEEKNEYLKR | parallel | < 15 °C |
| **P8SHb** | SPEDKIEELKRENRELERKIRELKRENEYLER | heterodimer |  |
| **P3S** | SPEDEIQQLEEEISQLEQKNSELKEKNQELKYGY | antiparallel | No data |
| **AP4S** | SPEDELQSNEEELQQNEQKLQQIKQKLQSIKYG | heterodimer |  |
| **P3** | SPEDEIQQLEEEIAQLEQKNAALKEKNQALKYG | parallel | 62 °C |
| **P4** | SPEDKIAQLKQKIQALKQENQQLEEENAALEYG | heterodimer |  |
| **GCN** | RMKQLEDKIEELLSKIYHLENEIARLKKLIGER | parallel homodimer | > 90 °C |


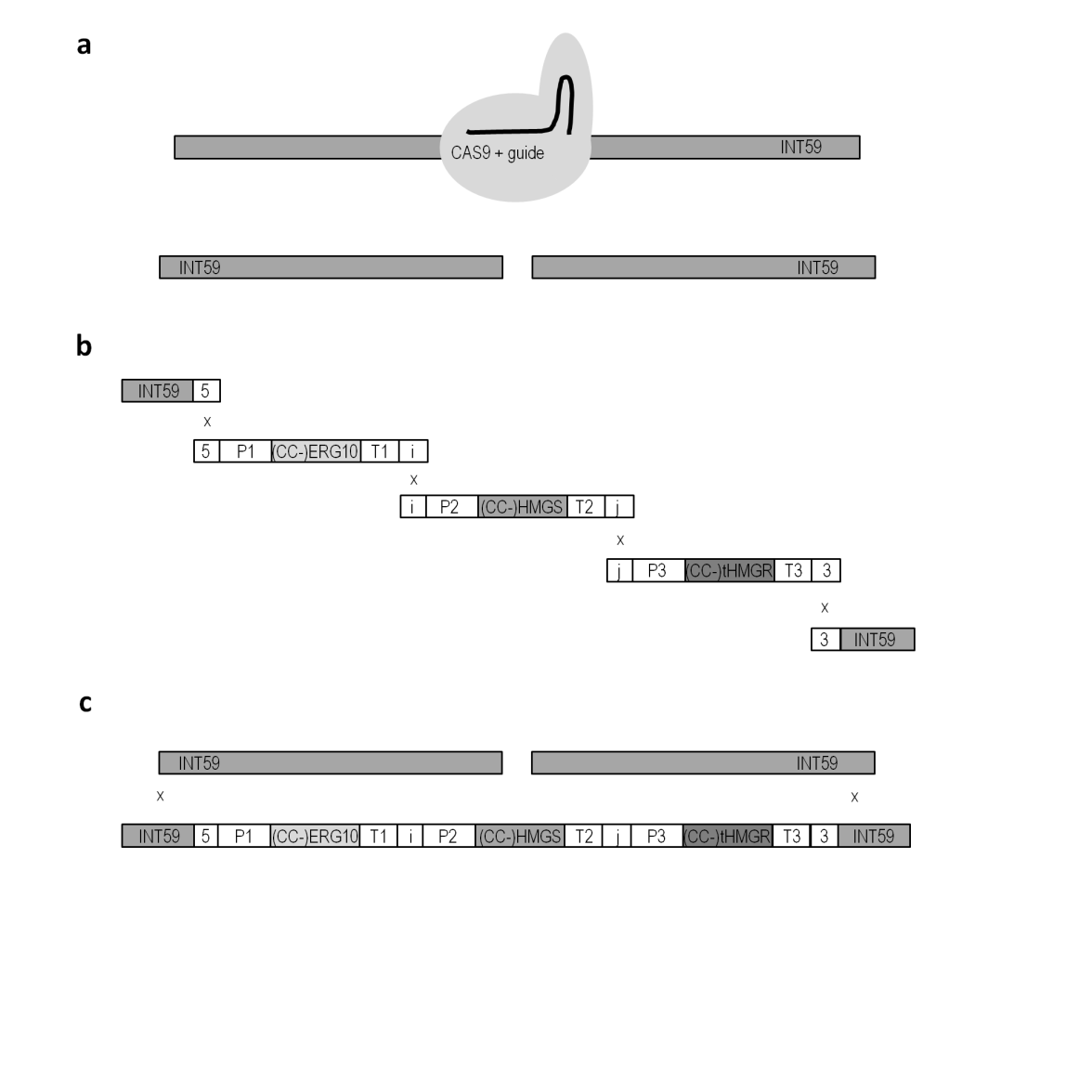


**Fig. S1** Introduction of mevalonate pathway donor DNA expression cassettes into the genomic DNA of *S. cerevisiae* using the experimental approach as described by Verwaal et al. (Verwaal et al. 2018). (**a**) Cas9 is directed to the intended genomic target site (sequence AGAAAACTCTTAGCTTTTCC) to create a double strand break. In the transformation mixture (**b**), donor DNA consisting of flank sequences and expression cassettes as well as carotenoid genes were included. **c** All donor DNA assembles into one stretch of DNA into genomic DNA around the INT59 locus (non-coding region between *SRP40* (*YKR092C*) and *PTR2* (*YKR093W*) located on chromosome XI) by *in vivo* recombination due to the presence of 50-bp homologous connector sequences, indicated as 5, i, j, or 3. P1-P3: different promoters (P1 *S. cerevisiae* PRE3p, P2 *K. lactis* TEF2p, P3 *K. lactis* TDH2p); T1-T3: different terminators (T1 *S. cerevisiae* TAL1t, T2 *S. cerevisiae* PDC1t, T3 *S. cerevisiae* TDH3t); CC - P3:GCN:P4, coiled-coil-forming domain.


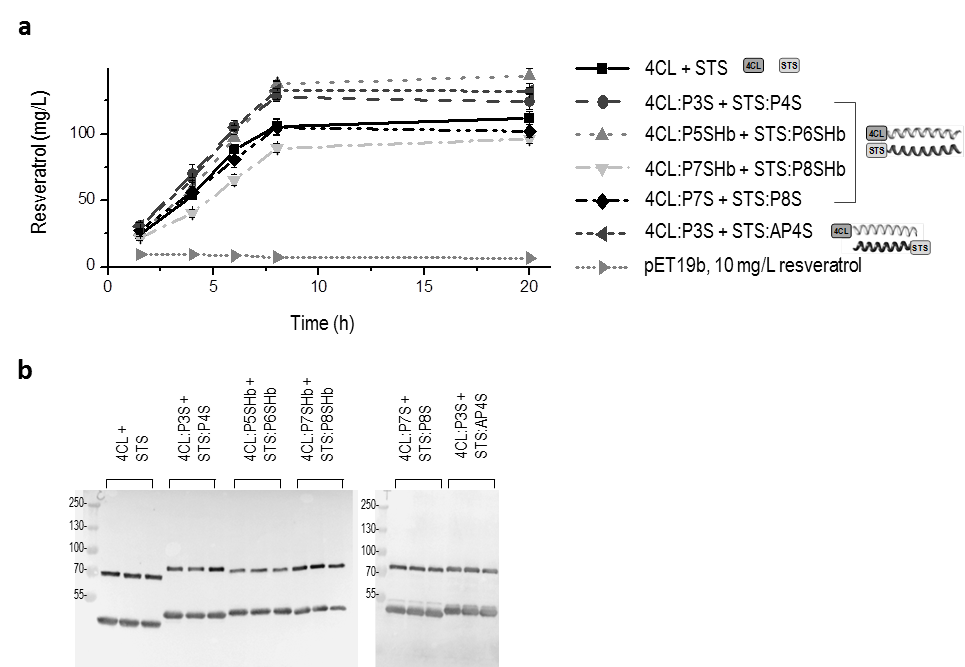


**Fig. S2** Formation of the enzyme clusters through coiled-coil-forming domains. (**a**) Enhancement of resveratrol biosynthesis in *E. coli* depending on the type of coiled-coil-forming domain fused to the enzymes. Enzymes were fused to several coiled coils with different stabilities and orientations. Simultaneously expressed native enzymes (4CL+STS) served as a reference. Values are the mean of three *E. coli* cultures ± standard deviation (s.d.) and are representative of three independent experiments. (**b**) At the time point 8 h, cells were lysed and the expression pattern of enzymes fused to different coiled coils in a soluble fraction was determined by Western blot. The data of three representative fermentations are shown.
